# Hib Vaccines: Past, Present, and Future Perspectives

**DOI:** 10.1155/2016/7203587

**Published:** 2016-01-20

**Authors:** Adi Essam Zarei, Hussein A. Almehdar, Elrashdy M. Redwan

**Affiliations:** ^1^Biological Sciences Department, Faculty of Science, King Abdulaziz University, P.O. Box 80203, Jeddah 21589, Saudi Arabia; ^2^Main Medical Laboratory, Medical Services, Saudi Airlines, P.O. Box 167, Cost Center 507, Jeddah 21231, Saudi Arabia; ^3^Therapeutic and Protective Proteins Laboratory, Protein Research Department, Genetic Engineering and Biotechnology Research Institute, City for Scientific Research and Technology Applications, New Borg EL-Arab, Alexandria 21934, Egypt

## Abstract

*Haemophilus influenzae* type b (Hib) causes many severe diseases, including epiglottitis, pneumonia, sepsis, and meningitis. In developed countries, the annual incidence of meningitis caused by bacteria is approximately 5–10 cases per population of 100,000. The Hib conjugate vaccine is considered protective and safe. Adjuvants, molecules that can enhance and/or regulate the fundamental immunogenicity of an antigen, comprise a wide range of diverse compounds. While earlier developments of adjuvants created effective products, there is still a need to create new generations, rationally designed based on recent discoveries in immunology, mainly in innate immunity. Many factors may play a role in the immunogenicity of Hib conjugate vaccines, such as the polysaccharides and proteins carrier used in vaccine construction, as well as the method of conjugation. A Hib conjugate vaccine has been constructed via chemical synthesis of a Hib saccharide antigen. Two models of carbohydrate-protein conjugate have been established, the single ended model (terminal amination-single method) and cross-linked lattice matrix (dual amination method). Increased knowledge in the fields of immunology, molecular biology, glycobiology, glycoimmunology, and the biology of infectious microorganisms has led to a dramatic increase in vaccine efficacy.

## 1. Introduction

Encapsulated* H. influenzae* type b (Hib) causes many severe infections, including sepsis, epiglottitis, pneumonia, and meningitis. Occasionally, encapsulated nontype b strains of* H. influenzae*, mostly type a, are able to produce invasive infections similar to Hib infections. In contrast, nontypeable (unencapsulated) strains are rarely a source of severe infections but most frequently generate infections of mucus membrane, such as conjunctivitis, sinusitis, and otitis media [1, 2]. Hib meningitis has been common in developed countries and hence must be presented along with the other Hib systemic diseases. In 1972, it was approximated that one in 280 newborns are infected with Hib in the first 5 years of life [3]. In a number of populations, including Australian aboriginal and Alaskan Eskimo children, incidence of meningitis caused by Hib has reached 1/50 to 1/30 newborns per year [4, 5]. The Hib meningitis mortality rate is about 5 to 10%, and around 30% of cured infected children have deficits of the central nervous system (CNS) varying from seizures, to deafness, to mental retardation [6]. Furthermore, Hib antibiotics resistance is increasing; around 30% of isolated Hib is ampicillin-resistant, and Hib meningitis in children is tenfold more transmissible than* Neisseria meningitidis* meningitis [7, 8]. In the prevaccination era, Hib epiglottitis caused much more morbidity and mortality than Hib meningitis and was second to Hib meningitis as the most common systemic Hib infection in Sweden [9].

According to the Global Alliance for Vaccines and Immunization (GAVI), more than 1.5 million children (around three per minute) die each year from diseases that could be prevented by vaccines. Enhancements in related fields such as biotechnology, virology, synthetic biology, and genetics offer a novel array of tools to advance vaccinology [10].

**Figure 1:**
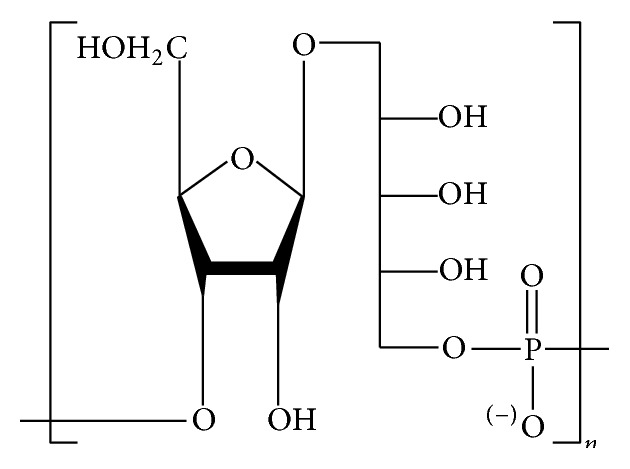
*H. influenzae* type b capsular polysaccharides repeating unit.

Capsular polysaccharide (CPS) covers the surface of some pathogenic bacteria, such as Hib, and is accessible for detection by cells of the immune system including macrophages, B cells, and dendritic cells. Moreover, most CPSs have unique structures that differ from those of mammalian glycans; and their accessibility by the immune cells and induction of immune responses specific for CPS make them excellent vaccine candidates [11]. The structure of the replicating disaccharide units of CPS of Hib is presented in Figure 1 [12]. These units are linked through phosphor-diester bonds, which generate the acidity of the polysaccharide Hib molecule [13].

The immunogenicity of CPS antigens leads to their categorization as T cell independent type 2 (TI-2) immune response, which stimulate protective antibodies without help from MHC-II classified T cells. CPSs trigger activation of the complement factor C3d by the complement alternative pathway; subsequently, primed marginal-zone B cells in the spleen travel to the germinal center and connect to polysaccharide-C3d via their CD21 complement receptor [14]. However, isotype switching follows, and responses against CPSs antigens occurred not only by IgM, but also by IgG and IgA [15, 16]. A specific signaling system might manage the vital responses of these antibodies [17], which differ in character and size among individuals [18] according to their age and earlier infections [19].

The naivety of the immune systems of young children and their relative incompetence compared to those of adults render children more vulnerable to Hib infections. Moreover, the integument and mucosa delicacy, as a kind of structural naivety, may play a role in susceptibility. Many studies have compared the immune systems of infants and adults, considering the role and potency of nearly every constituent of the immune system, humoral and cellular, innate and adaptive, that may make the infant immune system vulnerable [20]. The marginal zone of the human spleen is not completely formed until one to two years of age, numbers of CD21-expressing B cells in the marginal zone and complement are small at childbirth, and CPS-specific antibodies productions are limited or absent in newborns [21, 22]. At delivery, very little IL12 is produced from antigen presenting cells (APCs), and with the exception of live attenuated vaccines, which have a maturation effect on neonatal APC, most vaccines have little capability to prime protective T-helper 1 responses in newborns [23–28]. The suppression of antibody responses (mainly against protein antigens) in early life may be caused partly by transplacentally acquired IgG, which fades after birth according to a half-life of about 28 days; this transplacental IgG does not cause downregulation of T cell function [29].

When used as vaccines, CPS antigens of several infectious bacteria stimulate considerable protection by inducing antibody production [30]. Without considering herd immunity, the effectiveness of the conjugate Hib vaccine may range between a 46% and 93% reduction in invasive disease caused by Hib. The success and safety of the Hib conjugate vaccine have been confirmed in pharmacovigilance screening. Adverse reactions to the conjugate Hib vaccine are rare; only individuals with hypersensitivity to the vaccine's constituents are subject to contraindications [31]. However, children who contract Hib disease regardless of proper immunization must be examined for suspected malfunctions in their immune system that make them sensitive to the infection [32].

Several factors may explain the low introduction level of Hib conjugate vaccines in the majority of developing countries, such as the absence of statistics that reflect the burden of disease and the troubles facing its estimation and calculation, political failure to consider Hib infection a health crisis, and the absence of practices related to vaccinology [33, 34]. The use of smaller doses of antigen and smaller quantities for vaccine injection in developing countries could support the introduction of vaccines by immunization schedules such as those routinely planned for children [35].

The use of the Hib vaccine in Saudi Arabia began in April 1998 at King Fahad National Guard Hospital, where it was used routinely for all infants in the hospital [36]. Subsequently, vaccine became obligatory in the national immunization program in 2000 and since then cases of Hib have declined considerably. During three years (2001–2003) there were 30 cases of* H. Influenzae* invasive disease, compared to only 6 cases in the following three years (2004–2007) [37]. According to the Saudi Ministry of Health 2013 Statistics Book, coverage of the pentavalent vaccine (which includes diphtheria, pertussis, tetanus, Hib, and hepatitis B) is 97.7% [38]. This review on the vaccinology of Hib will serve as a valuable source of information for public health officials and decision-makers.

## 2. Hib Vaccines 

### 2.1. CPS Vaccines and Adjuvants

The idea of using the bacterial CPS in vaccines dates back to 1930s and involves inducing polysaccharide-specific antibodies to protect against pathogenic bacteria [39–41]. The CPS of Hib, polyribosylribitol phosphate (PRP, Figure 1), has several significant characters, such as immunogenicity in humans, the same antigenic properties in all strains of type b, and stimulation of anti-Hib antibodies. In 1985 purified PRP was certified for use in a vaccine in the United States. However, rapid reduction of antibodies stimulated by purified PRP was the main drawback, as it offered no immunological memory [42]. The solution was to give more injections as boosters that took dropping levels of antibodies back up to postvaccination levels, but this failed to generate either strong response nor immunological memory, as injection of the booster did not stimulate IgM class switching to IgG [43]. In Finland, the vaccine was very successful in experiments in children 18 months and older, while in the United States postmarketing surveillance showed fluctuating efficiency (from 69% to 88%) [44]. However, the level of ≥0.15 *μ*g/mL of anti-PRP in unvaccinated children and level of ≥1.0 *μ*g/mL in vaccinated children were considered protective against Hib infections, as suggested by many studies [45].

CPS-specific receptors on the surfaces of native B cells (BCRs) recognize the multivalency and large size of CPSs. Upon immunization with pure CPS, these BCRs bind to the CPSs and the BCR-CPS complex stimulates the B cell to induce and secrete IgM antibodies against CPS. In the event of bacterial infection following CPS immunization, IgM antibodies bind to the CPS expressed on the surface of the particular pathogenic bacteria and help eradicate that pathogen [11, 46].

The compounds that are able to enhance and/or regulate the antigen's immunogenicity are called adjuvants and represent a varied collection of compounds. Put simply, an adjuvant is a vaccine assistant (*adjuvare* is a Latin word meaning “to help”) and is a synonym of immunostimulant. It can be a combination of one or more compounds that act and function differently: that is, a molecule, carrier or depot, immunomodulator, and/or immunostimulant. The adjuvant industry has grown to meet demand for enhancing the immunogenicity of insufficient vaccines. The two main basic mechanisms by which adjuvants act are enhancing antigen presentation to the immune elements and stimulating immunity so that a stronger or wider response is reached. By these means, immunogenicity can be improved and/or doses of the vaccines can be decreased to a level that has no negative effect on immunogenicity [47, 48].

Animals, plants, and bacteria are sources of widespread immunostimulatory constituents of several adjuvants that function as stimulators of immunological components. One highly effective method of adjuvanticity is the conjugation of inactivated toxoids to purified PRP. This method has been used successfully in anti-Hib, anti-*N. meningitidis*, and anti-*Streptococcus pneumoniae* conjugate vaccines. Compounds such as alum and calcium phosphate are the only mineral salts authorized for use as adjuvants with vaccines in the United States since the 1920s. The primary mineral salt adjuvant for use in humans is alum, either as aluminum hydroxide or as aluminum phosphate. It acts by creating a depot spot, such that mild and constant release of the antigen improves presentation to immunological components [47, 48].

Studies have investigated the role of innate cells in adjusting adaptive immunity. Numerous antigen presenting cell (APC) signals are needed to start T-helper responses. First, a signal is activated by presentation of a certain peptide by class II molecules to the T cell receptor (TCR). Without the contribution of an additional signal, abortive and anergy responses are stimulated; a costimulatory second signal is required, via receptor-ligand contacts among APC/T antigens. It appears that another (preceding) signal, the zero signal, is required to stimulate APCs and orient subsequent Th responses, for example, throughout IL12 for Th1 responses. Zero signals are typically stimulated by detection of pathogen-associated molecular patterns (PAMPs) through pathogen recognition receptors (PRR), involving toll-like receptors (TLRs). APCs class I presentation for CD8 stimulation may be controlled by these signals. Promotion of CD4 cells that secrete interferon gamma (IFNg) by Th1 adjuvants is not enough to activate CD8 cytotoxic T cells, which also need class I antigen presentation [48]. These outcomes closely connect native immunity and the following adaptive immunity. The essential signals and steps needed to stimulate T and B cell responses suggest that signals of innate immunity also control the quality (not only the degree) of adaptive immunity. For example, innate cells permit adaptive T and B cells to differentiate and grow, and Th1 polarization is observed throughout IL12 secretion [48]. Adjuvants may be able to perform on any of these signals. Affecting exactly costimulatory molecules (second signal) throughout antibodies, chemokines, or cytokines is an attractive option [49], but it may lead to unfocused overreaction status [48].

While earlier development of adjuvants generated effective products, there is still a need to create new generations, rationally designed based on recent discoveries in immunology, especially innate immunity. The safe and optimal preparation of adjuvants is the most challenging task in the field. Little work has been dedicated to adjuvants with free PRP or with glycoconjugate vaccines, compared to protein vaccines. However, a number of researchers have examined the capacity of several molecules, especially TLR agonists, to increase the immunogenicity of conjugated or free carbohydrate antigens [48]. Free PRP vaccines against Hib are no longer used in the United States [50].

### 2.2. Conjugate Vaccines and Adjuvants

Most proteins need participation of T cells to stimulate antibody synthesis and consequently are considered T cell dependent (TD) antigens [51]. These proteins promote increased response of antibodies in the booster vaccine and encourage class switching from IgM to IgG via the involvement of T-helper cells in the immune process. Moreover, subsets of B-memory cells are generated from B cells in TD immunity, resulting in the establishment of memory against a specific antigen. This is accomplished by covalently connecting PRP to protein carriers, which results in production of Hib glycoconjugate vaccines [52]. By this kind of conjugation, the humoral immune response is induced with features of TD immunity reactions, such as affinity maturation, memory, and, crucially, the presence of immunogenicity in children aged more than 2 months [30]. Development of protein-conjugated polysaccharide vaccines has been accelerated by increasing antibiotic resistance and the need to stop Hib infections in high-risk populations [12].

In 1931, Avery and Goebel invented the improvement of polysaccharide immunogenicity by the tool of conjugation to proteins. Nevertheless, this was not widely practiced until the invention of polysaccharide conjugate vaccines. In the 1970s through 1980s, the application of bacterial polysaccharides and proteins for stimulating immunity increased. Conjugate Hib vaccines are created by Hib CPS (polyribosylribitol phosphate; 5-d-ribitol-(1→1)-*β*-d-ribose-3-phosphate; PRP) attached to proteins, with excellent safety and distinctly different structures and conformations [53–55]. The first invented and approved conjugate vaccine in the United States was created from PRP conjugated to diphtheria toxoid (PRP-D), which was withdrawn from the market soon after introduction of more effective vaccines using meningococcal outer membrane protein (PRP-OMP), CRM_197_ (PRP-CRM) protein carriers, or tetanus toxoid (PRP-T) [56].

The structure of polysaccharides and carrier proteins properties largely determine the immunogenicity of the conjugates, and the techniques used in the conjugation process can play a central effect. The conjugation process should be uncomplicated and mild and cause little alteration to the constituents, such that it does not damage important epitopes on either the protein or the PS, cause undesired depolymerization of the PS, or introduce any harmful epitopes. While a number of conjugation chemistries are available for the synthesis of PS-protein conjugates [57], only a few have been used in licensed vaccines. For proteins, surface-exposed amines (e.g., *ε* amines of lysine residues) and carboxyls (e.g., the side chains of carboxyl of aspartic and glutamic acid residues) are the major groups used for conjugation [58, 59]. Prior to the conjugation process, fermentation/isolation techniques of Hib CPS are used to produce Hib conjugate vaccines on a large scale [60].

**Figure 2:**
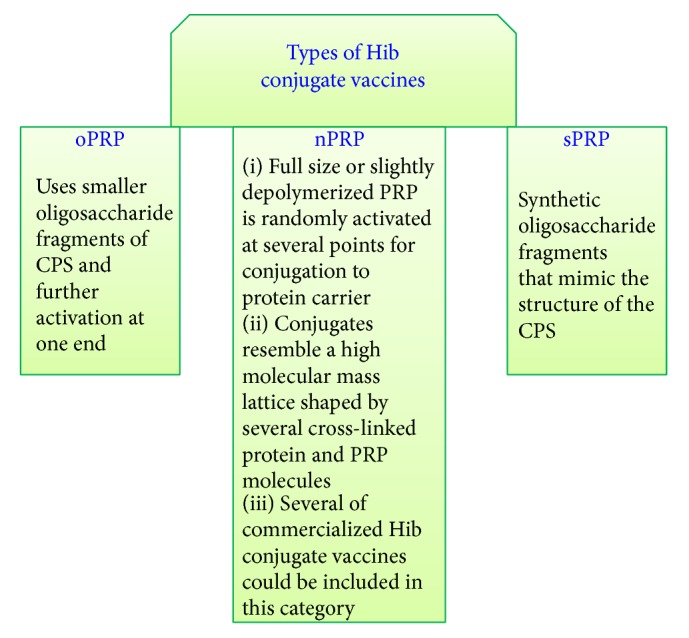
Schematic diagram summarizing the types of currently used Hib vaccines according to the type of PRP (nPRP-native PRP, oPRP-oligosaccharide PRP, and sPRP-synthetic PRP).

Chemical synthesis of the Hib saccharide antigen was a breakthrough in conjugated vaccines, which led to development of a novel type of Hib conjugate vaccine. This generally includes the formulation of the oligosaccharide moiety by chemical or enzymatic methods, which represents the immunological specificity for the vaccine and then attachment of this to an immunogenic protein by a linker. Chemical synthesis of these complex oligosaccharides is difficult and requires a suitable mixture of techniques that allow appropriate treatments by applicable glycosyl donors, acceptors, protecting groups, and coupling reactions settings. This option has come to be accessible due to notable growth of enzymatic and chemical oligosaccharides preparation [61–64]. Currently, the synthetic Quimi-Hib vaccine is approved for use in the National Immunization Program in Cuba [64].  Figure 2 summarizes the types of currently used Hib vaccines according to the type of PRP.

Glycoconjugate syntheses include the usage of well-defined or random activation sites in the polysaccharide as possible attachment sites for proteins. The choice of method for activation is mainly governed by the molecular size of the polysaccharide. Large polysaccharides are frequently arbitrarily activated, while selective activation at the reducing end is typical for oligosaccharides [12]. Molecular differences between conjugation methods do not affect their clinical performance but do influence strategies for quality control [57].

**Figure 3:**
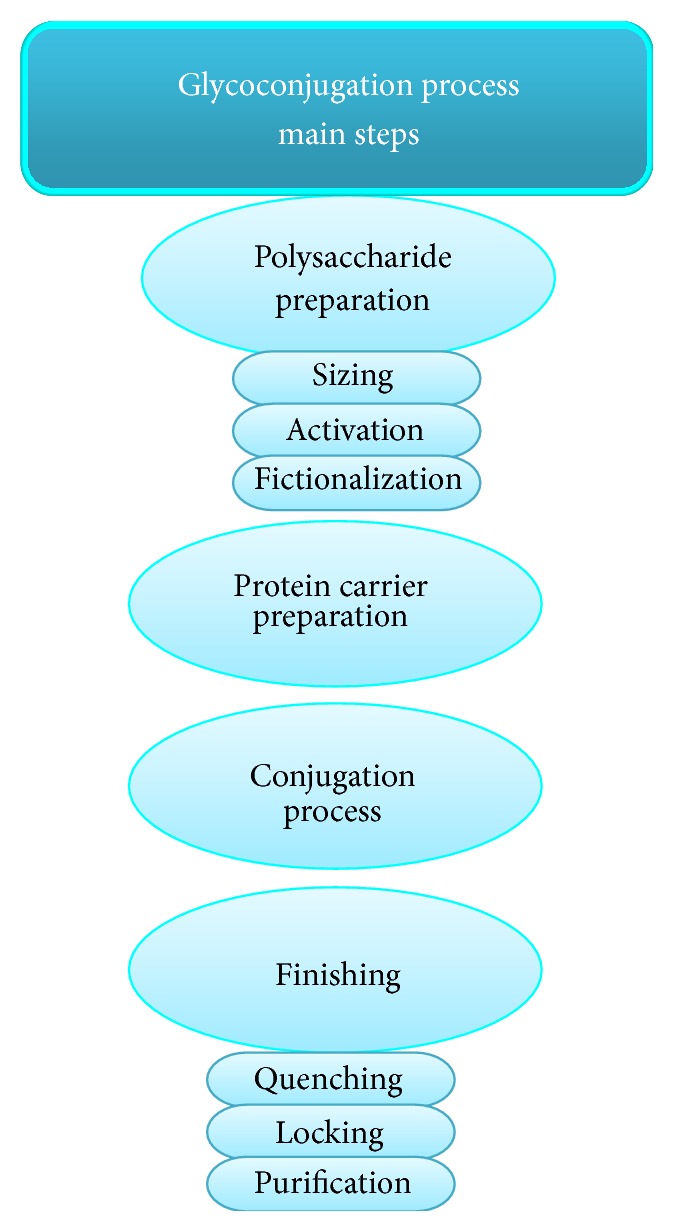
Main steps in the glycoconjugate process.

The conjugation process can be divided into the following steps (Figure 3): (I) preparing the carbohydrate, (II) preparing the protein carrier, (III) performing the conjugation, and (IV) finishing. Some conjugation schemes combine several steps, whereas in others a particular step may be unnecessary [58].

*Preparation of the Carbohydrate*

1. *Sizing*. Native CPSs have molecular sizes in the millions of Daltons, and solutions tend to be viscous. Reducing the molecular size of the PSs decreases their viscosity and makes the solutions easier to handle.
2. *Activation*. Carbohydrates have hydroxyl groups, which are relatively inactive and must be translated into a more reactive form (functional group). The activated PS can be either immediately conjugated to the protein or further functionalized. The positions of the activated groups determine the positions of the linkages on the PS.
3. *Functionalization*. This procedure adds reactive chemical groups to the carbohydrate or converts the activated PS into a more stable form while maintaining its reactivity. Functionalization can include the addition of a spacer molecule between the carbohydrate and the reactive group and in some cases is a multistep process [58].

*Protein Carrier Preparation*. Amines (the *ε* amines of lysines) and carboxyls (glutamic and aspartic acids) can be applied to link directly to the activated or functionalized carbohydrate. However, some protocols rely on the addition of chemical groups that are more reactive and/or more specific in their reaction with the activated or functionalized carbohydrate than carboxyls or amines. Functionalization of the protein can also provide a spacer molecule such that the reactive groups on the protein are more accessible to the carbohydrate [58]. 

*Conjugation*. Conditions that promote conjugation-high concentrations of protein and PS and high numbers of reactive groups bring the risk of excessive cross-linking when both the protein and the PS have multiple reactive groups. Also of concern is the need to confirm uniform mixing and reaction, which can be challenging on a production scale due to the high viscosity of the PSs. Careful control over factors relevant to the particular chemistry is crucial for successful conjugation. These factors include pH, temperature, the ratio of protein to PS, and the concentration of each. The type of linkage achieved depends on the chemistry and may be reversible or irreversible [58]. 

*Finishing*

1. *Quenching*. Quenching inactivates any residual groups to prevent further cross-linking. This step is commonly completed with monovalent blocking reagents, such as ethanolamine or glycine.
2. *Locking*. A locking (or blocking) step makes the conjugation linkage fundamentally irreversible.
3. *Purification*. The conjugate must be purified to eliminate the conjugation reagents and to guarantee low levels of unconjugated carbohydrate and protein. Unconjugated PS has been shown to decrease the immune response to the conjugate [65, 66].

Two models for carbohydrate-protein conjugation have been established: the single ended model (terminal amination-single method) and cross-linked lattice matrix (dual amination method) [12, 57].

#### 2.2.1. Terminal Amination-Single Method

**Figure 4:**
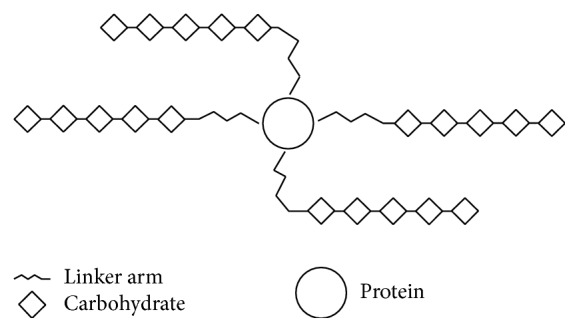
Single ended model (neoglycoprotein).

This method imitates the structural features of glycoproteins by which protein is connected to the oligosaccharides across its reducing end, resulting in the single ended model (Figure 4) [57]. The model differs from native glycoproteins in terms of polymeric carbohydrate chain length, connection sites, and structure. The antigenic carbohydrate positions of this model of neoglycoprotein are instantly available to antibodies. The carbohydrate hapten density is the main factor that influences the antigenicity of these neoglycoproteins [12].

#### 2.2.2. Dual Amination Method

**Figure 5:**
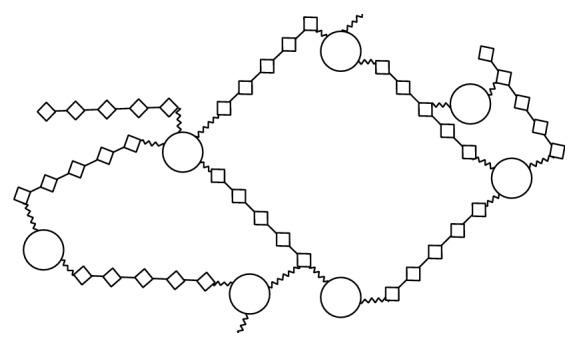
Cross-linked lattice model.

A cross-linked lattice matrix is molded by several connection points formed by conjugation of an antigenic carbohydrate and a protein carrier (Figure 5) [57]. Vaccine solubility can be enhanced by decreasing the quantity of cross-linked points on the polysaccharide chain, which shrinks the conjugate matrix and cross-linking. The large number of reachable antigenic spots on the superficial cross-linked lattice matrix guarantees the molecule's extraordinary immunogenicity, despite the quantity of cross-linking [12].

The tendency of the carrier protein to denature is a limiting factor for conjugation. However, immunogenicity can be maintained if an alteration in the tertiary geometric shape is able to influence the antibody specificity. The modification of hydroxyl, carboxyl, hemiacetal, phenoxyl, amino/imino, or mercapto/disulfide functional group is thus an important step in the conjugation process. Sodium cyanoborohydrate is used in conjugation chemistry involving reductive amination to selectively reduce intermediate imine adducts known as Schiff bases [67, 68]. This reduction pushes the system to equilibrium, affording stable adducts, despite the formation of Schiff bases being a disfavored equilibrium technique in water. Glycosylamine formation is straightforward reaction whereby carbohydrates are treated with saturated solution of ammonium bicarbonate for 5–7 days at room temperature. Catching the glycosyl amine by creation of the peptide with iodoacetic acid and reaction of the cysteine residue of the protein to the thiol reactive iodoacyl group produces neoglycoconjugates [69–71]. The spacer arm, an allyl group presented at the reducing end, may be employed to make aldehyde by ozonolysis and can be joined to the protein amine groups by reductive amination.

A thiol reactive maleimide group can be connected to a carbohydrate over a spacer arm to increase the availability of functional groups and reactive centers [72]. Methods using N,N′-dicyclohexylcarbodiimide (DCC), water soluble 1-ethyl-3-(3-dimethylaminopropyl) carbodiimide (EDC), or activation of carboxylic groups by sulfo-NHS for combined amino functionality in a carbohydrate or protein have been broadly applied to the production of neoglycoproteins [73, 74].* p*-Nitrophenyl glycosides may be converted to highly reactive diazonium salts to create electron-rich adducts, aromatic tyrosine, or tryptophan residues [75]. Although regarded as limited by recent measures, diazo coupling grants strong conjugates immunogenicity and has been broadly utilized for preparation of conjugate vaccines such as the type 3* S. pneumoniae* CPS vaccine. By nonspecific activation of hydroxyl groups and creation of reactive cyanate esters, cyanogen bromide has been employed to conjugate carbohydrates and protein amino groups in aqueous alkaline solution via a stable* O*-alkyl isourea linkage [76]. When a phosphate presents at the terminal end, it frequently appears in an internal ester, because of the closeness of the adjoining hydroxyl group on the ribitol moiety to the phosphate hydroxyl; this internal esterification triggers Hib PS hydrolytic lability at both alkaline and acid pH and at elevated temperature [77].

Adjuvants for conjugate vaccines include salts of aluminum. Depending on the conjugate, the adjuvant may be unimportant or crucial [78]; or it may have a harmful influence on the stability of the conjugate, as with Hib conjugates vaccines [79]. Toll-like receptor 9 (TLR9) agonists have also been employed as adjuvants, helping to compensate for immunodeficiency such as that faced in late or early life [80–82]. Immune interference arising in polyvalent conjugate vaccines could be lessened by adjuvants via suppression of the B or T cell dependent carrier, among other methods [83–85].

Quality control of Hib conjugate vaccines by laboratories, manufacturers, and the government depends on physicochemical procedures for screening of production consistency and recognition of any defects in batches over time. Biological tests are performed by manufacturers only during vaccine production to confirm stimulation of TD immunity by conjugate vaccines and to guarantee their safety [86].

Since 1997, the World Health Organization (WHO) has recommended the Hib conjugate vaccine for national immunization programs anywhere resources exist and disease burden data support its priority [39, 87–89]. Before 1997, however, only a minority of children in developing countries had access to the Hib conjugate vaccine, due to shortages arising from sparse statistics on disease burden [90], lack of belief in the vaccination value, lack of awareness, and greater priority of other concerns [91]. The vaccine was supplied by only one manufacturer to the United Nations International Children's Emergency Fund (UNICEF) [92], and its three-dose course was much more expensive than the combined price of all other vaccines in the standard immunization for infants specified by the WHO Expanded Program on Immunization (EPI). The estimated price of the Hib conjugate vaccine was twofold higher than the measles, mumps, and rubella multivalent vaccine, and it was more costly than conventional vaccines in EPI (3.15 versus US$1.4/dose) [93]. Because of a lack of competition, the vaccine price remained high for several years [92]. The vaccine formulation choice (monovalent or multivalent) (Table 1) and final product vialing (1- or 10-dose vial) were crucial factors in the introduction of the Hib vaccine in North and South America. These two factors affect the vaccine wastage level, cost, reconstitution necessity, space of cold chain, and education events for medical employees [94, 95].

The regimen of three doses in the first year of life, with a fourth dose given in the second year, is followed in majority of the world. A three-dose regimen without the fourth dose is typical in low-income countries. Generally the Hib conjugate vaccine is given as a one-injection polyvalent together with tetanus toxoid, diphtheria toxoid, and whole-cell or acellular pertussis (DTwP or DTaP), and sometimes additionally with hepatitis B antigen (Table 1) and/or inactivated poliovirus [47].

In the 1990s patent law prevented vaccine manufacturers in developing countries from acquiring the techniques for making Hib conjugates [96, 97]; and markets were cautious due to a lack of dependable estimation. Additionally, manufacturers in developed countries began cancelling conventional manufacture of DTwP vaccines, which were less costly and hence obtainable to UNICEF for developing countries [98]. To deal with these obstacles, the National Institute for Public Health and the Environment/Netherlands Vaccine Institute (RIVM/NVI, Bilthoven, Netherlands) initiated a plan in 1998 to develop commercially viable and scalable Hib conjugate vaccine production, without patent violation, by employing technology transmissible to manufacturers in developing countries. Further assistance for the growth of combined vaccines containing the Hib conjugate indicated that manufacturers, by acquisition of entrance to technology for the Hib conjugate vaccine, could subsequently confirm a sustainable supply of inexpensive and valuable vaccines [98].

Founded in 2000, the Developing Countries Vaccine Manufactures Network (DCVMN) was formed to distribute surveillance against identified and emergent contagious infections, with the goal of raising the obtainability and quality of vaccines inexpensive to everyone. DCVMN is an international alliance of manufacturers that provides data and professional education plans, development of technology, inspiring transfer of technologies, advanced research and development, and community teaching regarding the accessibility of secure, cheap, and effective vaccines. The network grew to involve 44 manufacturers in 16 territories and countries, creating and distributing >40 several vaccine types [99]. Arabio, founded in 2005, was the first biopharmaceutical company in the Gulf Cooperation Council (GCC), located in Jeddah BioCity with Vacsera as a limited liability company, and is a member of DCVMN. Arabio focuses on plasma, biopharmaceutical, and vaccine products. The range of developments planned by Arabio could establish it as the leading biological company of its category in the Middle East [100].

## 3. Future Perspectives

**Figure 6:**
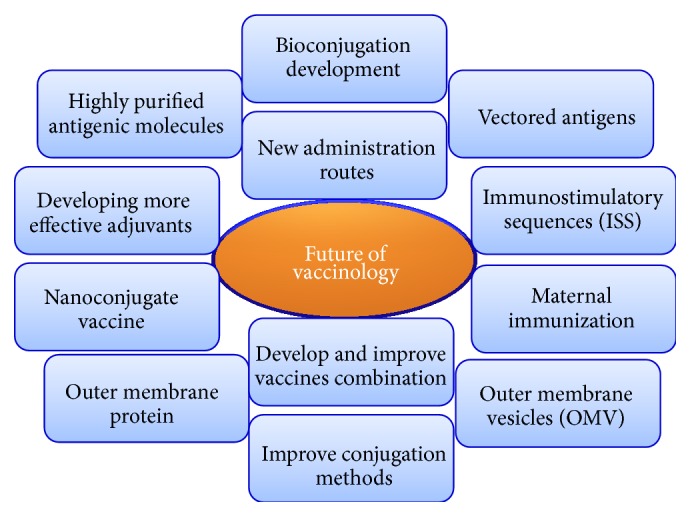
The most important fields in the future of vaccinology as a whole and Hib vaccines in particular.

The immune and central nervous systems are characterized by their ability to retain memory. This exceptional quality creates many opportunities for health interventions, such as prophylactic immunization. Advances in molecular chemistry and biology, glycobiology, glycoimmunology, pathogen biology, and immunology have led to substantial increases in vaccine efficacy. Current vaccines are commonly made from highly purified antigens or hapten obtained from or designed on the most immunogenic parts of pathogens. The latest molecular biology advances also promote techniques helping in immune system stimulation [12].  Figure 6 summarizes the most important fields in the future of vaccinology as whole and Hib vaccines in particular. In addition, new vaccinology concentrates on tools that offer sustained immunogenicity with better safety. These technologies consist of employing engineered antigens, subunits from pathogens (polysaccharides or proteins), or vectored antigens. The vector is a harmless virus or bacterium in which a gene can be enclosed and expressed. This technique has come to dominate the field due to its usefulness for hard-to-reach pathogens, such as intracellular pathogens [101].

Vaccinology is further improving via development of more adjuvants, combination vaccines, and new methods of administration. New administration methods include transcutaneous (intradermal) immunization (TCI), which has potential for use in humans, whereby antigens are transported by Langerhans antigen presenting cells to nearby lymph nodes, directly stimulating systemic immunity. A major model for this method is the intradermal influenza vaccine, which penetrates the epidermis with a microneedle and creates the same immune responses as intramuscular vaccines [101, 102]. Moreover, via an ADP-ribosylating exotoxin in mouse, such as the heat-labile enterotoxin of* Escherichia coli *or its mutants (LTK63 and LTR72) or cholera toxin, TCI activates antitoxin protecting responses and the coadministered antigen [103–108].

Combination vaccines are increasingly significant for efforts to improve vaccination acceptance by the public, as they reduce the number of injections essential for complete immunization (Table 1). Combined vaccines help to achieve high vaccination coverage and well-timed vaccination by minimizing occurrences of delay. Including novel antigens on current high-coverage vaccines is an effective method for presenting novel antigens to schedules of vaccination [109]. Studies conducted in Germany [110] and the United States [39, 111] confirm that combined vaccines can lead to improved coverage of specific antigens, and with further well-timed vaccination, a greater proportion of children can obtain all recommended vaccines at the appropriate age [112].

In Germany, 5-year surveillance was conducted to inspect the efficiency of hexavalent vaccines and found that DTPa-HBV-IPV/Hib was 90.4% effective for a complete three-dose primary series and 100% for a complete primary series with a booster (regardless of the priming Hib vaccine) [113]. Long-standing effectiveness of a combined Hib-DTPa-based vaccine on a 3-, 5-, and 11-month schedule has also been confirmed in Sweden, as incidence in children younger than 4 years was approximately 0.4/100,000 in between 2005 and 2008 [114].

Maternal immunization to protect from pathogenic organisms has been suggested as a technique to protect newborns, as maternofetal immunoglobulin transfer occurs through the placenta and maternal immunoglobulins appear in the fetus blood. Maternal immunization with Hib polysaccharide and conjugate vaccines appears to be a successful approach for delivering antibodies protective levels to infants. Maternal immunization studies [115, 116] have recognized several factors affecting the placental passage of immunoglobulins. Compared to IgM and IgA antibodies, IgG antibodies more effectively cross the placenta, and antibodies in the IgGl subclass are better for transport than those in the IgG2 subclass [117]. Antibody placental crossing is moreover subject to the vaccination time through pregnancy [116]. The detection of factors affecting antibody placental crossing may be fundamental to choosing plans for future maternal immunization [118].

Synthetic oligodeoxynucleotides enclosing cytosine triphosphate deoxynucleotide-phospho-diester-guanine triphosphate deoxynucleotide (CpG) immunostimulatory sequences (ISS) may function as danger signals of bacterial attacks and trigger immune warfare [119, 120]. While the exact mechanism of action is not fully understood, it is thought that ISS act as PAMPs able to connect PRR, mainly expressed on the surface of antigen nonspecific innate immune cells [121]. The presence of APC-expressed PRR by PAMPs activates innate cells that gain the power to stimulate adaptive immunity cells, which in turn support the launch of an antigen-specific immune action. ISS are made in accordance with DNA sequences of bacteria that may stimulate innate immunity cells [122]. ISS stimulate innate immunity in the same way as bacterial infections, therefore supporting the specific immunity to the antigen, in contrast to other adjuvants, such as mineral oils or alum, whose immunostimulatory action extends delivery of antigens [120]. The danger signals produced by ISS provide a powerful adjuvant to stimulate the anti-conjugated PS type one immunity response.

The adjuvant effect of ISS effectively improves the anti-carrier immunity response. Anti-diphtheria toxoid (anti-DT) and anti-tetanus toxoid (anti-TT) antibody titers were greatly improved by ISS in the Hib-CRM and Hib-TT vaccines. Moreover, the pattern of the anti-carrier IgG subclass was affected by ISS, as IgG2a and IgG2b increased in the presence of ISS. A considerable rise in IgG3 was distinguished only in the anti-TT immune response. IgG3 is the major IgG subclass in several humoral immunity responses in the mouse that are stimulated by polysaccharide antigens but is a minor constituent of the anti-protein immune response [123]. Therefore, TT but not CRM stimulates IgG3 production in the similar cytokine milieu resulting from ISS, supporting the suggestion that the antigen structure is included in the immunoglobulin isotype switch of B-lymphocyte differentiation [124].

*H. influenzae *outer membrane proteins have received attention due to their antigenic excellence as potential vaccine candidates [125]. However, one significant criterion that must be met for a Hib outer membrane protein to be an effective vaccine is that the protein must have surface-exposed and antibody-accessible antigenic determinants that are common to most strains of the pathogen. At least three Hib outer membrane proteins appear to fulfill this requirement. Data on the P6 protein show that this protein has at least one surface epitope that is common to all strains [126]. To date, P2 and P6 are the best candidates for anti-nontypeable* H. influenza* vaccines [127]. Another method in vaccine development inserts surface-exposed proteins in outer membrane vesicles (OMV), which, in clinical trials, stimulates responses of serum bactericidal antibodies and gives rise to antimeningococcal activity [128].

### 3.1. Conjugation Development

The classic antigen presentation hypothesis for glycoconjugate vaccines suggests that helper CD4^+^ T cells identify a peptide originated from the carrier protein [129]. According to the traditional pattern, the glycoconjugate binds to the surface of a B cell whose specific job is to make antibodies to the polysaccharide component. The B cell deals with the protein portion of the glycoconjugate and exhibits a peptide from the covalently linked carrier protein in the setting of the major histocompatibility complex class II (MHC II) to the *αβ*-TCR of CD4^+^ T-helper cells. Stimulation of T cells by peptide-MHC II complexes and other costimulatory molecules leads to production of the cytokine interleukin 4 (IL-4) by the T cell, which in sequence encourages cognate B cell maturation and subsequent creation of carbohydrate-specific antibodies, with associated class switching of immunoglobulin to IgG and memory responses. This hypothesis was originally based on the theory that only protein antigens can be presented to and identified by T cells. Initial research on hapten-carrier conjugates (i.e., small molecular weight noncarbohydrate molecules connected to carrier proteins) suggested that peptide presentation is the triggering tool for glycoconjugate induced T cell activation [130].

**Figure 7:**
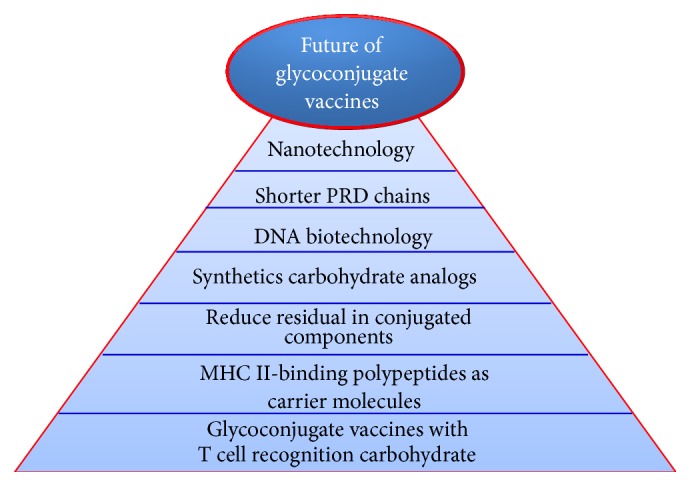
The most important approaches to improve glycoconjugate vaccines.

A new study [129] strongly suggests that carbohydrate-recognizing T cells, not peptide-recognizing T cells, are the principal T cell population driving the T cell-mediated adaptive immune response. It shows that carbohydrates, which were previously thought to be “T cell-independent” antigens, can be precisely distinguished by T cells, as they are conjugated with carrier peptides that permit their presentation by MHC II molecules on the surface of APCs. These conclusions are different from the classical understanding that T cells can only recognize peptides [129]. A model new-generation glycoconjugate vaccine [129] has enriched carbohydrate T cell epitopes compared to a glycoconjugate vaccine made by traditional conjugation methods. This model vaccine is 50–100 times more immunogenic than classical vaccines. This type of carbohydrate-recognition T cell may lead to significantly better vaccines against infectious diseases. Elucidation of the T cell epitopes of glycoconjugate vaccines would allow us to imitate those epitopes and enhance vaccines by joining carbohydrate epitopes at a frequency and density that enhances immunogenicity and protection through APCs proficient processing and presentation and highly specific T cell recognition [131]. The zwitterionic polysaccharides are a class of complex carbohydrates that activate T cells [131, 132].  Figure 7 summarizes the most important approaches to the improvement of glycoconjugate vaccines.

The conjugation of biomolecules to metal nanoclusters and nanoemulsions has opened new opportunities in the design and synthesis of multifunctional and multimodal built systems for biomedical uses. Gold nanoparticles have been widely studied for their low toxicity, relative inertness, easy handling, and easy chemistry of surface control. The surface of gold can be shaped in a controlled way with various ligands through thiol chemistry, producing multifunctional and multivalent nanoparticles [101, 133].

Carrier proteins are fundamental components of classical conjugate vaccines. Peptides offer covalently bound carbohydrate epitopes to the TCR of CD4^+^ T cells [134]. Thus, optimization of the MHC II-binding peptide concentration in a conjugate vaccine would theoretically enhance the immunogenicity of the conjugate vaccine by increasing the number of carbohydrates that can be presented on MHC II proteins. The best approach to accomplish this is to connect polysaccharides to MHC II-binding peptides in place of the intact proteins. This would allow the presentation of significantly greater amounts of peptide-bound carbohydrate epitopes to T cells, compared to when intact proteins are. In fact, many studies have shown that conjugating peptides to polysaccharides creates highly effective conjugate vaccines [134–137]. In one study, three polypeptides consisting of strings of 6, 10, or 19 human MHC II-binding peptides from several antigenic proteins were created [138]. In each construct, the MHC II-binding peptides were separated by the lysine-glycine spacer to give flexibility to the polypeptide and to permit the conjugation of the glycan to the amino side chains of the lysine spacer. The 19-valent polypeptide was then conjugated with CPSs of* N. meningitides* serogroups A, C, W-135, and Y [139]. In immunization tests, the polypeptide-containing glycoconjugate vaccines had greater immunogenicity than glycoconjugates that contained CRM_197_ as carrier protein. Compared to CRM_197_-based conjugates, smaller quantities of polypeptide-based conjugates stimulated a higher bactericidal antibody titer against all four meningococcal polysaccharides. This strategy illustrates the use of MHC II-binding polypeptides as carrier molecules to create greatly effective glycoconjugate vaccines. The main improvement enabled by this strategy is that these synthetic polypeptide carriers can be identified by most of the human MHCII haplotypes. Furthermore, peptide carriers can be expressed in* E. coli*, which makes them easy to produce for production of glycoconjugate vaccines [11].

An important step in conjugation chemistry is to improve conjugation efficiencies to levels at which residual unconjugated components, particularly free PSs, do not affect the stimulation of protective immune responses. The use of an effective, mild conjugation chemistry would allow for higher yields of vaccine. For example, reaching more than 90% efficiency of coupling of the PS may allow for a simplified purification process. It would also be needed for development of conjugate vaccines that do not require refrigeration [58].

Current improvements in carbohydrate synthesis must simplify the enzymatic and chemical preparation of well-characterized and well-defined homogenous carbohydrate antigens for further development of glycoconjugate vaccines. Several carbohydrate antigens analogs that are stable metabolically, such as S-glycosides and C-glycosides, may enhance the immunogenicity and antigenicity of the glycoconjugate vaccines. Moreover, such analogs should expand our knowledge of antibody carbohydrate identification, processing and presentation of antigens, and immune system stimulation. Such improved knowledge of glycoimmunology would facilitate the creation of the ideal glycoconjugate vaccine for a particular disease or infection. Synthetic C-glycoside analogs of tumor-combined antigens, which are metabolically stable, nonhydrolysable carbohydrates, would similarly enhance batch-to-batch consistency in vaccine manufacturing and bypass the cold storage that is generally needed to preserve and guarantee effectiveness of existing carbohydrate-based vaccines [12].

Recently, a study reported highly immunogenic PRP-TT conjugates generated from shorter chain PRP, compared to both their high-molecular-weight counterparts from the same laboratory and licensed vaccines. The authors have optimized methods to prepare more immunogenic low-molecular-weight PRP-TT conjugates in a reproducible manner. The higher reactivity of hydrazide groups compared to the lysine *ε*-amino group resulted in a shorter conjugation time and an optimal yield. The conjugates thus created are immunogenic in rats. A hallmark of this study is the development of highly immunogenic PRP-TT conjugates using short-chain PRP, which forms the basis for further development of oligosaccharide conjugates as successful vaccine candidates alone or in combination [140].

### 3.2. Conjugation through DNA Biotechnology

Generally, chemical approaches that allow for well-controlled conjugation reactions between distinct-length glycans and proteins [141–143] are vital for designing highly immunogenic vaccines. To date, the most effective vaccines used for protection from invasive pathogenic bacterial infection are the glycoconjugate vaccines, mainly consisting of bacterial capsular polysaccharides coupling to a desired protein carrier. Within the pathogenic bacterial strains there is effective single CPS component and/or multiple CPS from different strains. The later condition makes glycoconjugate vaccine production a very hard process, since each strain requires extraction, hydrolysis, chemical activation, and conjugation to a carrier protein. To avoid this hard situation for conserved and/or nonconserved CPS of different bacterial pathogen, the use of bioglycoconjugates machinery of innovative* E. coli* will substantially simplify the production of glycoconjugate vaccines.

DNA biotechnology has revolutionized vaccinology and generated new methods for vaccine discovery, such as antigenome technology, reverse vaccinology, surfome analysis, genetics vaccinology, and immunoproteomics to uncover innovative immunogenic antigens [144]. Genetically engineered vaccines are expected to be a major type of future vaccines due to their high safety level, improved immunogenicity, and decreased reactogenicity. However, absence of posttranslational modifications (PTMs) machinery in* E. coli* limits its use for the production of recombinant biopharmaceuticals and/or biosimilars [145–152]. Various posttranslational modifications including glycosylation and phosphorylation, which are critical for functional activity, do not take place in* E. coli *[149–153]. Protein glycan coupling technology (PGCT) is a strategy that has been used to move toward the target of making structurally defined, homogenous glycoconjugate vaccines with a well-controlled conjugation reaction [154–156]. N-linked glycosylation of proteins is one of the most important posttranslational modifications in eukaryotes. Kowarik et al. identified a novel N-linked glycosylation pathway in the bacterium* Campylobacter jejuni* and also showed the successful transfer of functionally active N-glycosylation pathway into* E. coli* [157]. PGCT uses the bacterial glycosyltransferase enzyme Pg1B, an oligotransferase expressed in* Campylobacter jejuni*, to enzymatically link a bacterial polysaccharide with a carrier protein. Pg1B specifically recognizes a 2-acetamido-containing a reducing end sugar and glycosidically links it to a carrier protein that has a specific sequence identified by the Pg1B enzyme for N-linked glycosidation. The enzymatic conjugation, and glycan and protein biosynthesis, occurs in an* E. coli* host strain. The target glycan, protein, and Pg1B are cloned and expressed in* E. coli*. This technology removes the necessity for multistep production and purification of the glycan and protein and several steps of chemical conjugation. Consequently, the process of creating glycoconjugate vaccines is shortened and vaccine production is enhanced. Furthermore, current chemical conjugation strategies require alteration of the polysaccharides or proteins (e.g., random oxidation of the sugar chain), which may change the natural epitopes and lead to generation of less-effective conjugates. Additionally, enzymatic conjugation uses highly specific substrates with distinct binding domains, and as a result, the conjugation outcomes are homogenous and structurally defined. Finally, PGCT avoids the need for pathogenic bacteria from which polysaccharides and/or proteins are isolated, as both glycan and protein synthesis and glycoconjugate assembly take place completely in an* E. coli* system [156].

Although the bacterial N-glycan structure is not similar to that seen in eukaryotes, engineering of glycosylation pathway of* C. jejuni *into* E. coli* has not paved the way for expressing and production of glycosylated protein* E. coli* [158, 159], but the glycoconjugate vaccines too [154, 156, 160–164], which will avoid the chemical conjugation vaccines producers for the harsh capsular polysaccharide-protein conjugation chemical reactions. The coming challenge for synthesis of glycosylated proteins in the engineering* E. coli* is the robotic machinery for secretory and extracellular N-linked glycoproteins production [165].

## 4. Conclusion

Heterogeneity in antibody responses between individuals and racial groups is controlled by genetic allotypes, which are still only partly understood and may be well-defined for governing T-independent and T-dependent antibody responses in certain circumstances. Moreover, as immunity weakens with age and fluctuates due to previous pathogen contact, life-cycle controlling vaccines implemented for several periods of life can make the most of defense at all ages. The recent concept of “one vaccine suits everyone” to immunize individuals is the only practical concept at the current time, but it is possible that future vaccines will be developed according to national boundaries and personalized to each individual beneficiary; this is termed “vaccinomics.” Sustainment of surveillance of colonization and isolates from diseases will be a vital part of a planned strategy for immunization in coming years.

Several populations remain vulnerable to Hib disease even with vaccination. Native American populations are up to six times more susceptible to Hib infection, and Alaskan natives and Navajo Indians still get Hib disease. Studies in Europe and the United States raise the question of whether three primary injections and one boost at around 15 months of age are sufficient, or if something more is needed to protect these populations, especially if the number or people around them who carry* H. influenzae* or other bacteria will not give them a boost in immunity. It has long been established that vaccine manufacturers in developing countries are able to manufacture high quality new vaccines that can satisfy WHO requisites. The Hib conjugate vaccine RIVM/NVI can offer an effective prototype for the efficacious resettlement of technology of up-to-date vaccine to the Arab Gulf and developing nations, including Saudi Arabia.

## Figures and Tables

**Table 1:** Summary of the most important Hib vaccines in U.S. Food and Drug Administration (FDA) and The European Medicines Agency (EMA) up to 20 December 2015.

Vaccine name	Approval date	Valency	Description	Manufacture and marketing
b-CAPSA 1 Hib-VAX Hib-IMUNE	1985 US	Monovalent	Pure polysaccharide CPS vaccine (unconjugated). The vaccine was recommended routinely for children at 24 months of age and for children at 15 months of age enrolled in child care facilities. The vaccine was not consistently immunogenic in children <18 months of age and was withdrawn from the market in 1988.	b-CAPSA 1 by Praxis, Biologics, Hib-VAX by Connaught, and Hib-IMUNE by Lederle

ProHIBIT	1987 US	Monovalent	First conjugate *Haemophilusinfluenzae* b vaccine to be approved by FDA. ProHIBIT is no longer available in the United States.	Connaught Laboratories, Inc.

HibTITER	1990 US	Monovalent	Hib conjugate vaccine (diphtheria CRM197 protein conjugate). Discontinued 2007.	Wyeth Pharmaceuticals Inc.

PedvaxHIB	1989 US	Monovalent	Hib conjugate vaccine (meningococcal protein conjugate).	Merck & Co., Inc. http://www.merck.com/product/vaccines/home.html

ActHIB	1993 US	Monovalent	Approved for use as a four-dose series in infants and children 2 months through 5 years of age.	Sanofi Pasteur http://www.sanofipasteur.com/en/

OmniHib	1993 US	Monovalent	Hib conjugate vaccine. No longer available in the United States.	SmithKline Beecham

TriHIBit	1996 US	Polyvalent	Combination of DTaP and Hib vaccine, licensed for the fourth dose in the DTaP and Hib series. Discontinued on 2011 according to CDC.	Sanofi Pasteur http://www.sanofipasteur.com/en/

Comvax	1996 US	Polyvalent	Mixed vaccines of recombinant hepatitis B antigen and *Haemophilusinfluenzae* type b conjugated vaccine. Discontinued on 2014 according to Merck.	Merck & Co., Inc. http://www.merck.com/product/vaccines/home.html

Tetramune	1996 US	Polyvalent	DTP and Hib conjugate vaccine. Discontinued according to CDC.	Wyeth Pharmaceuticals Inc.

PROCOMVAX	1999 EU	Polyvalent	Hib conjugate and hepatitis B (recombinant) vaccine. Withdrawn on 2009 according to EMA.	Sanofi Pasteur MSD http://www.spmsd.com/

Hexavac	2000 EU	Polyvalent	Diphtheria, tetanus, acellular pertussis, inactivated poliomyelitis, hepatitis B (recombinant), and Hib conjugate vaccine, adjuvant. Suspended 2005, withdrawn on 2012 according to EMA.	Sanofi Pasteur http://www.sanofipasteur.com/en/

Infanrix-Hib	2000 EU 2002 US	Polyvalent	DTaP and adsorbed conjugated Hib vaccine.	GlaxoSmithKline Biologicals https://www.gsk.com/

Infanrix Hexa	2000 EU	Polyvalent	DTaP, hepatitis B (recombinant), inactivated poliomyelitis, and adsorbed conjugated Hib vaccine.	GlaxoSmithKline Biologicals https://www.gsk.com/

Quintanrix	2005 EU	Polyvalent	DTP, hepatitis B (rDNA), and adsorbed Hib conjugate vaccine. Withdrawn on 2008 according to EMA.	GlaxoSmithKline Biologicals https://www.gsk.com/

Pentacel	2008 US	Polyvalent	DTaP adsorbed, inactivated poliovirus, and Hib conjugate.	Sanofi Pasteur http://www.sanofipasteur.com/en/

Hiberix	2009 US	Monovalent	Hib conjugate vaccine (tetanus toxoid conjugate). Approved only as a booster dose of the Hib schedule among children 12 months and older.	GlaxoSmithKline Biologicals https://www.gsk.com/

MenHibrix	2012 US	Polyvalent	Meningococcal groups C and Y and Hib tetanus toxoid conjugate vaccine for infants at increased risk of meningococcal disease.	GlaxoSmithKline Biologicals https://www.gsk.com/

Hexacima	2013 EU	Polyvalent	DTaP, hepatitis B (rDNA), poliomyelitis (inactivated), and adsorbed Hib conjugate vaccine.	Sanofi Pasteur http://www.sanofipasteur.com/en/

Hexyon	2013 EU	Polyvalent	DTaP, hepatitis B (rDNA), inactivated poliomyelitis, and Hib conjugate vaccine (adsorbed).	Sanofi Pasteur http://www.sanofipasteur.com/en/

INFANRIX-IPV/Hib	2015 US	Polyvalent	DTaP, inactivated poliomyelitis, and Hib vaccine.	GlaxoSmithKline Biologicals https://www.gsk.com/

CRM197: enzymatically inactive and nontoxic form of diphtheria toxin that contains a single amino acid substitution.

DTaP: diphtheria, tetanus, and acellular pertussis.

DTP: Diphtheria and tetanus toxoids and whole-cell pertussis.

CDC discontinued vaccines:

http://www.cdc.gov/vaccines/pubs/pinkbook/downloads/appendices/B/discontinued_vaccines.pdf.

CDC, Morbidity and Mortality Weekly Report (MMWR):

http://www.cdc.gov/mmwr/preview/mmwrhtml/00000696.htm.

EMA, European public assessment reports:

http://www.ema.europa.eu/ema/index.jsp?curl=pages/medicines/landing/epar_search.jsp&mid=WC0b01ac058001d124.

MERCK, COMVAX vaccines discontinue report:

https://www.merckvaccines.com/is-bin/intershop.static/WFS/Merck-MerckVaccines-Site/Merck-MerckVaccines/en_US/Professional-Resources/Documents/announcements/VACC-1114028-0000.pdf.
